# The presence of SARS-CoV-2 in multiple clinical specimens of a fatal case of COVID-19: a case report

**DOI:** 10.1186/s13256-022-03706-y

**Published:** 2022-12-22

**Authors:** Liba Sokolovska, Anna Terentjeva-Decuka, Maksims Cistjakovs, Zaiga Nora-Krukle, Sabine Gravelsina, Anda Vilmane, Katrine Vecvagare, Modra Murovska

**Affiliations:** Institute of Microbiology and Virology, Riga Stradins University, Riga, 1067 Latvia

**Keywords:** SARS-CoV-2, COVID-19, Severe disease, Lymphoma, Viremia, PBMC

## Abstract

**Background:**

The risk of developing severe and even fatal coronavirus disease 2019 (COVID-19) increases with various factors such as advanced age and chronic diseases, especially those treated with immunosuppressive drugs. Viral ribonucleic acid (RNA) and viral load detection in extra-pulmonary specimens have been proposed to indicate disease severity.

**Case presentation:**

Here we describe a fatal COVID-19 case of an 83-year-old Caucasian male patient with various underlying comorbidities, including cardiovascular and autoimmune disorders, as well as immunosuppression due to lymphoma treatment. Upon admission, the patient was radiologically diagnosed with severe COVID-19. The patient was febrile and presented with diarrhea, continued dyspnea, tachypnea, and low blood oxygen saturation, treated with high-concentration oxygen supplementation and antibacterial therapy. Overall the patient was treated for COVID-19 for 19 days. Blood tests were performed upon admission, on the fifth, 10th, 13th, and 19th day. In addition, nasopharyngeal swab, blood, urine, and fecal samples were collected from the patient on the 14th day for virological and immunological investigations. Severe acute respiratory syndrome coronavirus 2 (SARS-CoV-2) was detectable in all samples collected from this patient, including blood plasma and peripheral blood mononuclear cells (PBMC), with very high viral loads. However, neither virus-specific IgA, IgM, nor IgG antibodies were detectable.

**Conclusions:**

The various cardiovascular, autoimmune, and oncological disorders, advanced age, and the high levels of inflammatory markers predisposed the patient to severe COVID-19 and determined the fatal outcome of the disease. We believe that the multiple specimen SARS-CoV-2 positivity and extremely high viral loads in nasopharyngeal swab and fecal samples to be the result of COVID-19 severity, the inability of viral clearance and weakened immune response due to advanced age, comorbidities, and the presence of non-Hodgkin's lymphoma and the immunosuppressive treatment for it, highlighting the risks of COVID-19 in such patients.

## Background

The coronavirus disease 2019 (COVID-19) pandemic has caused a global health crisis, especially endangering the elderly and people with chronic illnesses. A combination of these two and other risk factors predisposes individuals to clinically severe COVID-19 and increases the risk of a fatal outcome.

Several studies have identified certain factors indicative of disease severity and mortality risk, such as advanced age, underlying comorbidities such as heart disease and oncological diseases, laboratory parameters such as lymphopenia, high levels of lactate dehydrogenase, procalcitonin, C-reactive protein (CRP) and several inflammatory cytokines such as Interleukin-6 (IL-6) [1, 2]. Some reports have also pointed to the increased load of severe acute respiratory syndrome coronavirus 2 (SARS-CoV-2) and the prolonged infectivity of patients with severe COVID-19 [3].

Here we describe a fatal case of an 83-year-old COVID-19 patient exhibiting several high-risk predisposing factors using clinical data and immunological and virological investigations of multiple specimens obtained from this patient during hospitalization.

## Case presentation

**Fig. 1:**
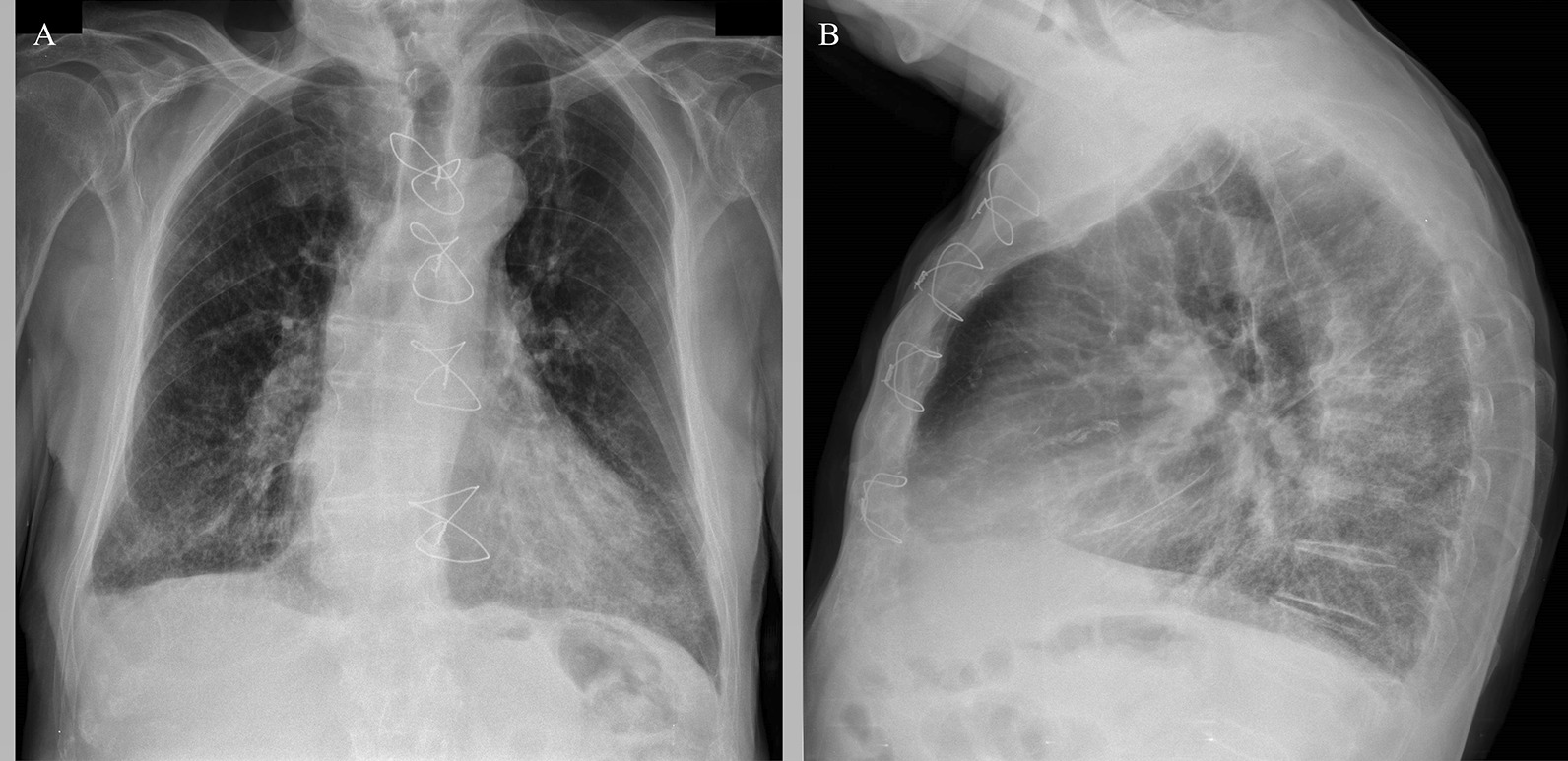
Chest X-ray images obtained upon admission to LCID. Chest X-ray findings included infiltrations in the lower fields of both lungs, cardiomegaly, Kerley B lines, upper lobe pulmonary venous congestion, right pleural effusion. Bilateral basal pneumonia and heart failure X-ray signs. **A** Posterior-anterior chest X-ray; **B** Lateral view chest X-ray

An 83-year-old Caucasian male was hospitalized at a local hospital for erysipelas on both legs. Upon admission, the patient was routinely tested for COVID-19 by a reverse transcriptase-polymerase chain reaction test and was found to be positive. The patient was not vaccinated against COVID-19. While in the hospital, the patient did not develop any COVID-19-related symptoms. After receiving treatment for erysipelas for seven days, the patient was stable and was discharged. While at home, COVID-19 symptoms—fever, cough, weakness, and diarrhea appeared, gradually worsened, and 13 days after the onset of symptoms, the patient was admitted to the Latvian Centre of Infectious Diseases (LCID). Upon admission to LCID, the patient did not display any neurological problems as contact with the patient could be established, and he could orient in time and space. The skin and mucous membranes were pale and dry, with signs of recurrent erysipelas on both legs. During auscultation, diffuse crackles on both sides of the lungs were found, and palpation of the abdomen did not show any pathology, including no signs of dysuria; the patient had mild peripheral edema on both legs. The patient had a febrile body temperature of 38.0 ºC, tachycardia with a heart rate of 100/min, blood pressure was 110/70 mmHg, and respiratory rate was 24/min with blood oxygen saturation of 89% on room air. The patient was diagnosed with clinically severe COVID-19 with bilateral pneumonia, as determined with radiological imaging (Fig. 1), and respiratory failure type I [4].

From the second to the fourth day of hospitalization, as antibacterial and symptomatic treatment was started, the patient's body temperature remained subfebrile with cough, watery stool 3–4 times a day, dyspnea, and tachypnea with low blood oxygen saturation (93%), supplemented with 4–6 L of oxygen per minute through a nasal cannula.

On the fifth day of hospitalization at LCID, a *Clostridium difficile* infection was confirmed*.* Patient fecal samples were collected according to the hospital's guidelines, and tests for *C. difficile* glutamate dehydrogenase and *C. difficile* toxin A/B were performed*.*

From the sixth to the ninth day of hospitalization, the patient remained stable but required oxygen supplementation; the diarrhea had resolved by day seven.

On the tenth day of hospitalization, the patient presented with recurrent febrile body temperature of 38.5–39.0 ºC, severe malaise, disorientation, and blood oxygen desaturation. In addition, laboratory findings showed significantly elevated inflammatory markers, and the decision was made to switch to antibacterial therapy and to start oxygen supplementation through a high-concentration oxygen mask with a reservoir bag of 15 L per minute.

From the eleventh to the fourteenth day of hospitalization, the patient's medical condition stabilized yet remained severe, showing little to no improvement. From day 15 patient's dyspnea worsened, and the blood oxygen saturation fell to 88% despite the oxygen supplementation. Oxygen supplementation was continued through a high-concentration oxygen mask with a reservoir bag of 25 L per minute, with some improvements to blood oxygen saturation (92–93%).

Gradually the patient's condition worsened with no improvements, despite the treatments. The patient died from COVID-19-associated lung damage and respiratory failure on November 2020 after being treated for 19 days. An autopsy was not performed at the request of the patient's relatives.

The patient had a history of cardiovascular problems (coronary heart disease, myocardial infarction, followed by percutaneous coronary intervention with stent implantation, permanent atrial fibrillation, stable angina pectoris) and a myriad of other chronic health problems, including non-Hodgkin's lymphoma, melanoma, lymphostasis in both legs after erysipelas, vitiligo, benign prostatic hyperplasia. Diffuse large B cell lymphoma was histologically diagnosed in June 2020, and since October, the patient was treated with rituximab. For the lymphoma treatment patient also took methylprednisolone 4 mg per os (p/o) once per day, allopurinole 300 mg p/o once per day, and trimethoprim-sulfamethoxazole 400–80 mg p/o twice a week. Before hospital admission, the patient was treated for his cardiovascular diseases with dabigatran 110 mg p/o twice a day, torasemide 10 mg p/o once a day, and atorvastatin 20 mg p/o once a day.

The patient was a non-smoker and had no history of alcohol abuse. The patient lived in a countryside house with his oldest son. The patient was retired, and no information on past employment or the patient's family history could not be obtained from the patient.

At LCID, the patient was treated with oxygen through a nasal cannula (4–6 L per minute) for 9 days, and a high-concentration oxygen mask with a reservoir bag (15–25 L per minute) for 10 days, dexamethasone 8 mg intravenously (i/v) once daily for 19 days, dual antibacterial therapy—ceftriaxone 2000 mg i/v once daily for 12 days and doxycycline 100 mg p/o twice a day for 11 days, then switching to piperacillin/tazobactam 4000 mg/500 mg i/v three times a day for nine days, enoxaparin-sodium 0.4 mL subcutaneously once daily for 19 days, metoprolol 50 mg p/o once daily for 19 days, torasemide 5 mg p/o once daily nine days, spironolactone 25 mg p/o once daily for 11 days, omeprazole 20 mg p/o once daily for 19 days, metamizole 1000 mg i/v in case of febrile temperature, and troxerutin 2% gel for local use for 16 days. On the day the patient died, he received methylprednisolone 1000 mg i/v and morphine 1%—3 ml i/v.The patient did not receive any antiviral drugs because he did not meet the criteria needed. The patient did not receive monoclonal antibodies or convalescent plasma because at the time the patient was hospitalized, these medications were unavailable in the country.

The patient's blood was tested upon admission to LCID, on the 5th, 10th, 13th, and 19th day of hospitalization. Nasopharyngeal swab, blood, urine, and fecal samples were collected from the patient on the 14th day.

Blood test results are illustrated in Table 1. The parameters analyzed differed between the tests. Analysis of circulating blood cells revealed that the patient had elevated leukocyte count since day 10, reaching 14.96 × 10^3^ cells/µL on day 19. Upon admission, the neutrophil count was normal (6.13 × 10^3^ cells/µL, ref. range 2.0–7.0 × 10^3^/µL) but then increased and stayed elevated since day 10. Upon admission, lymphocyte count was significantly decreased (0.23 × 10^3^ cells/µL, ref. range 1.2–3.5 × 10^3^/µL) falling to 0.06 on day 19. Several markers of inflammation and infection were found to be elevated. Procalcitonin levels were high upon admission, but decreased (while still being higher then normal) by day five after being treated (0.61 vs 0.24, ref. range 0.00–0.05). IL-6 was also severely elevated (93.0 pg/mL, ref. range 0.0–3.4 pg/mL). CRP levels were elevated in all of the time-points, reaching levels as high as 239.3 mg/L on the day the patient died.

**Table 1:** Blood test results done upon admission, on the 5th, 10th, 13th, and 19th day of hospitalization

	Reference range	Upon admission	5th day	10th day	13th day	19th day
Leukocytes (µL)	4.0–9.0 × 10^3^	6.88	–	11.00	11.56	14.96
Red blood cells (µL)	4.0–5.5 × 10^3^	2.94	–	3.04	3.12	3.22
Hemoglobin (g/dL)	13.0–16.5 × 10^3^	9.10	–	8.90	9.10	10.60
Hematocrit (%)	40–48	31.40	–	28.30	29.20	33.50
Platelets (µL)	150–400 × 10^3^	102.00	–	195.00	256.00	244.00
Neutrophils (µL)	2.0–7.0 × 10^3^	6.13	–	10.53	10.86	14.71
Banded Neutrophils (%)	1–6	–	–	14	6	46
Segmented Neutrophils (%)	40–72	–	–	79	88	47
Lymphocytes (µL)	1.2–3.5 × 10^3^	0.23	–	0.12	0.11	0.06
Monocytes (µL)	0.2–0.8 × 10^3^	0.30	–	0.32	0.56	0.16
Eosinophils (µL)	0.04–0.5 × 10^3^	0.03	–	0	0	0
PCT (ng/mL)	0.00–0.05	0.61	0.24	–	–	–
ALAT (U/L)	< 41	17.07	25.00	–	–	10.00
ASAT (U/L)	< 40	35.91	–	–	–	–
GGT (U/L)	< 61	–	111.00	–	–	–
Bilirubin (µmol/L)	< 21	13.51	–	–	–	–
Potassium (mmol/L)	3.5–5.1	3.51	4.75	–	–	3.93
Sodium (mmol/L)	136–146	130.31	137.50	–	–	141.50
Creatinine (µmol/L)	62–106	88.26	70.00	–	–	125.00
GFR (MDRD, mL/min)	> 90	75.92	100.00	–	–	51.00
CRP (mg/L)	< 5	166.70	41.70	177.00	50.00	239.30
D-dimer (μg/mL)	< 0.5	1.44	1.45	–	–	–
IL-6 (pg/mL)	0.0–3.4	–	93.00	–	–	–
Troponin T (pg/mL)	< 50	89.03	–	–	–	–
HIV test		–	NEG	–	–	–

*PCT* procalcitonin, *ALAT* alanine aminotransferase, *ASAT* aspartate aminotransferase, *GGT* gamma-glutamyl transferase, *GFR* glomerular filtration rate, *MDRD* modification of diet in renal disease equation, *CRP* C-reactive protein, *IL-6* interleukin 6, *HIV* human immunodeficiency virus

Blood plasma and peripheral blood mononuclear cells (PBMC) were isolated immediately upon receiving the peripheral blood samples. PBMCs were isolated using HISTOPAQUE by Sigma (United Kingdom) gradient. RNA was isolated from all samples obtained using the Ribospin vRD kit by GeneAll (South Korea) according to the manufacturer's instructions (adapted specifically for fecal samples). SARS-CoV-2 was detected using the Direct SARS-CoV-2 Realtime PCR kit by Vircell (Spain), and the viral load was determined using the quanty COVID-19 kit by Clonit (Italy).

**Fig. 2:**
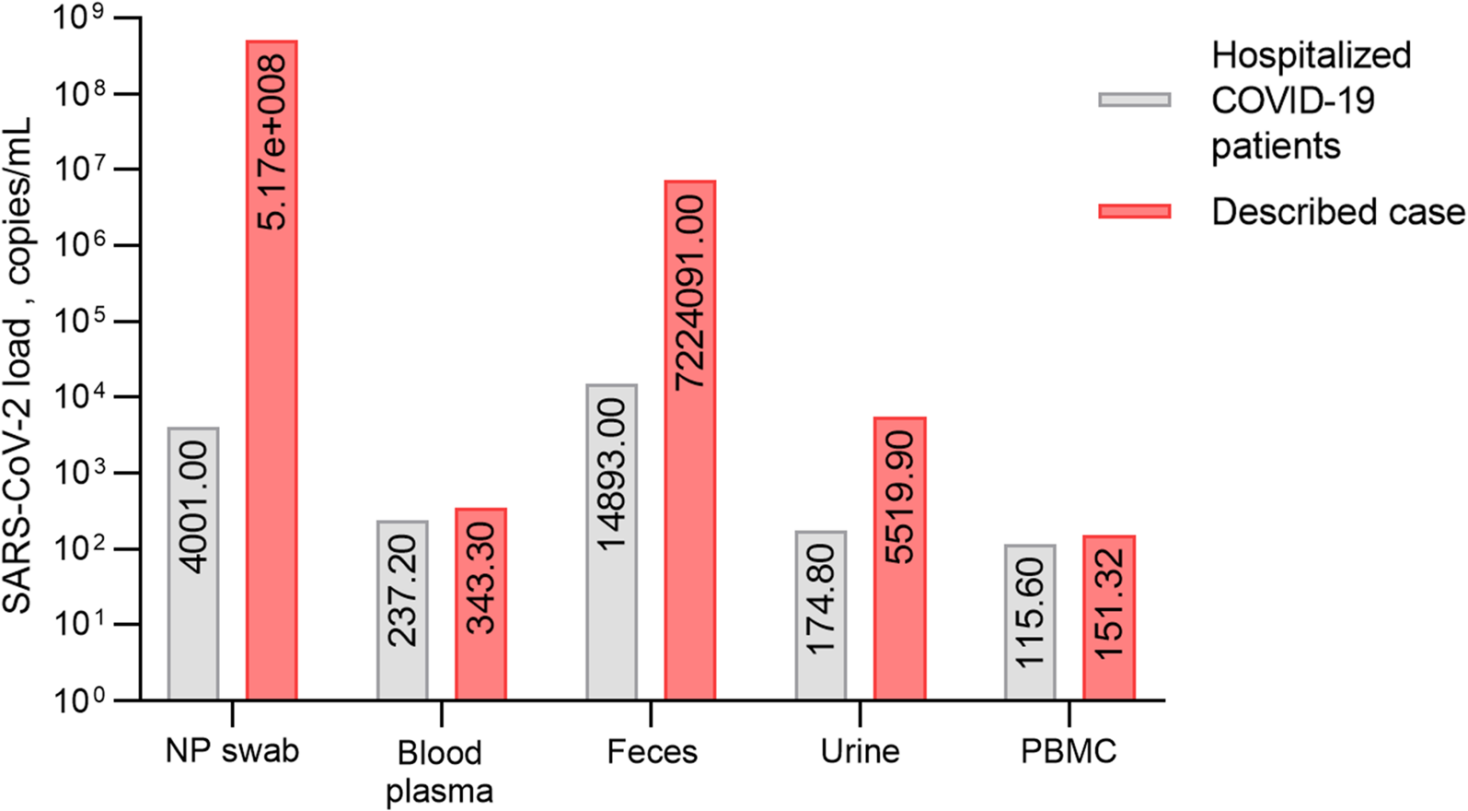
SARS-CoV-2 load in various clinical samples obtained from the described case compared to the SARS-CoV-2 load determined in samples obtained from 139 hospitalized COVID-19 patients (unpublished data). *NP swab* nasopharyngeal swab

SARS CoV-2 genomic sequence was detected in all biological samples analyzed—nasopharyngeal swab, plasma, PBMCs, urine, and feces. As expected, the highest viral load was observed in the nasopharyngeal swab sample (517,000,000.0 viral copies/mL), but the viral load in feces was also notable. We believe that the detected loads illustrate an extreme case. As these patient samples were collected as a part of a larger research project, we could compare the determined viral loads with other hospitalized COVID-19 patients. The viral load of the described patient's fecal sample was significantly higher when compared to the median viral loads of the fecal samples obtained from 139 hospitalized individuals—7,224,091 vs 14,164 copies/mL (*p* < 0.0001), and an even more pronounced difference was observed in the case of the nasopharyngeal swab load—517,000,000 vs 5752 copies/mL (*p* < 0.0001) (unpublished data). Interestingly SARS-CoV-2 sequence was detectable in both cell-free blood plasma and PBMCs (Fig. 2).

Plasma samples were used to semi-quantitatively detect SARS-CoV-2 specific (nucleocapsid protein (NCP) and spike protein subunit 1 (S1)) IgA and IgG class antibodies using Anti-SARS-CoV-2 ELISA by Euroimmun (Germany), to quantitatively detect SARS-CoV-2 specific IgM and IgG class antibodies using Anti-SARS-CoV-2 ELISA by Antibodies-online (Germany), and to determine the levels of inflammatory cytokines using custom multiplex assay by Merck Millipore (Germany). Neither quantitative nor semi-quantitative analysis of SARS-CoV-2 antibodies detected antibody titers, indicating seronegativity.

Levels of 14 cytokines were analyzed—granulocyte–macrophage colony-stimulating factor (GM-CSF), interferon-gamma (IFN-γ), interleukin 1β (IL-1β), interleukin 6 (IL-6), interleukin 8 (IL-8), interleukin 17A (IL-17A), interleukin 18 (IL-18), interferon gamma-induced protein 10 (IP-10), monocyte chemotactic protein 1 (MCP-1), macrophage inflammatory protein-1 alpha and beta (MIP-1α/β), platelet-derived growth factor (PDGF-AB/BB), tumor necrosis factor-alpha (TNF-α) and vascular endothelial growth factor A (VEGF-A). Levels of 5 of the analyzed cytokines—GM-CSF (< 2.6), IFN-γ (< 1.3), IL-1β (< 1.6), IL-17A (< 1.3), and MIP-1α (< 3.0), were below the detection limit of the test utilized, even though detectable and elevated levels of these cytokines could be found in a group of other hospitalized COVID-19 patients. The described patient exhibited severely elevated levels in comparison to median levels of other hospitalized patients, for example, in the case of IL-6 (20.2 vs 4.9 pg/mL), IL-18 (105.2 vs 53.6 pg/mL), and IP-10 (2007.5 vs 639.2 pg/mL), respectively. On the other hand, the level of PDGF-AB/BB was significantly lower in the patient's plasma when compared to the median levels of other hospitalized patients—1399.0 vs 25,606.0 pg/mL (unpublished data).

## Discussion

Chronic health problems are known to be implicated in the risk of suffering from severe COVID-19; in this patient's case, the risk was heightened not just by one but by several co-morbidities. Here we have described a case of severe COVID-19 of a high-risk patient harboring high SARS-CoV-2 loads in various samples collected from the patient during hospitalization, while not developing any SARS-CoV-2 specific antibodies—neither IgG, nor IgM. SARS-CoV-2 loads were detected in nasopharyngeal swab, fecal, urine, blood plasma and most intriguingly—PBMC samples, suggesting potential viral replication in these cells and its role in immune dysregulation and lymphopenia, as other researchers have suggested [5] and overall immune response impairment**.** This multiple comorbidity-influenced predisposition, combined with the patient's advanced age and widespread detection of viral RNA, determined the severe and fatal outcome of the SARS-CoV-2 infection.

Many studies have explored the increased risk of severe and even fatal COVID-19 due to comorbidities, advanced age, and other factors. The odds of developing severe COVID-19 and the likelihood of a fatal outcome have been shown to increase with age. A study from China, where data from almost 80 000 confirmed cases was used, demonstrated that patients over 59 years were 5.1 times more likely to die after developing symptoms [3, 6]. A seroepidemiological study from Spain reported that the infection fatality risk increased rapidly after age 50 and ranged from 11.6 to 16.4% in men aged 80 years and older [7].

Comorbidities, especially cardiovascular disorders, have been highlighted as risk-heightening since the early months of the pandemic [8]. The above-reported case also supports this, as one of our patient's comorbidities was coronary heart disease, which has been shown to increase the odds of in-hospital death substantially and is more present in COVID-19 non-survivors [9, 10].

As the pandemic progressed, more and more comorbidities joined the list of risk factors for severe or fatal COVID-19, many of which the described patient possessed, oncological disorders being one of them. Non-Hodgkin's lymphoma, more precisely B-cell hematological malignancies, are frequently treated with anti-CD20 monoclonal antibodies, such as rituximab, which rapidly deplete the numbers of mature CD20-positive B-cells—both normal and malignant. While treating the malignancy, B-cell depletion disrupts the production of antibodies, thus hindering the humoral memory and immune response to new pathogens and predisposing patients to infections [11]. Studies have demonstrated that patients with B-cell malignancies recently treated with anti-CD20 medications and patients older than 70 years have prolonged in-hospital stay and show higher mortality from COVID-19 [12, 13]. The described case illustrates all of the risks associated with this malignancy in the context of COVID-19—prolonged in-hospital stay, the fatality of the disease, and as the antibody level investigations revealed—impaired humoral immune response as neither SARS-CoV-2-specific IgM, IgA nor IgG class antibodies were detected.

Laboratory parameters highlighted by other studies [1] were also applicable in the case described above, as lymphopenia, elevated white blood cell count, increased levels of CRP procalcitonin, D-dimer, and IL-6 could be observed. CRP was high in all of the blood tests of our patient. Initial elevation of CRP levels may be partly due to erysipelas, and the second spike *Clostridium difficile* infection and the progression of lung damage. Several studies have evaluated these parameters as predictors of disease severity. In the case of CRP, levels upon admission have been shown to be significant predictors of disease severity [14]. A study determined that patients with CRP levels higher than 41.8 mg/L were at increased risk of severe COVID-19 [15].

Cytokine storm has been well established as a driver of COVID-19 pathology and tissue damage. Such cytokines as IL-6, IL-10, TNF-α, IL-2, MCP-1, and others have been involved in this inflammatory phenomenon [16] and were investigated in the patient's plasma. While some demonstrated the signature elevated levels, several of the cytokines implicated in COVID-19-associated cytokine storm were below the detection limit in this patient. For example, in the case of PDGF-AB/BB, detectable levels were found but were more than ten times lower when compared to other hospitalized patients, even though other studies have demonstrated higher levels in severe cases [17]. Interestingly, the levels of GM-CSF were below detection. GM-CSF is a player in hyperinflammation if present in excess, but also can confer lung protection as illustrated by mouse studies and studies on patients with acute respiratory distress syndrome, where early elevated expression of GM-CSF correlated with increased survival [18, 19]. The undetecatable levels of GM-CSF in the patient may point to another aspect of impaired immune response against the infection.

The unique combination of risk factors observed in this case, combined with abnormal blood cell counts and detectable SARS-CoV-2 genomic sequence in PBMCs, may have disturbed immune cell functionality, including cytokine signaling, resulting in the differences in cytokine levels observed when comparing to other hospitalized COVID-19 patients who had no detectable SARS-CoV-2 in their PBMCs.

Detection of SARS-CoV-2 in PBMCs has been described before, even after the Severe acute respiratory syndrome coronavirus (SARS-CoV) epidemic [20, 21]. Several studies have demonstrated the capability of SARS-CoV-2 to infect blood cells in vitro [5, 22, 23]. Even though the virus can be found in PBMCs, especially in more severe cases of COVID-19, the clinical relevance remains to be elucidated. It is unclear whether SARS-CoV-2 in PBMCs is a driver or a consequence of disease severity, but it has been shown to further increase inflammation in COVID-19 patients [23, 24].

Several studies have identified the load of SARS-CoV-2 as a predictor of more severe disease and mortality. Higher viral loads were found in severe COVID-19 cases, some showing 60 times higher viral loads [25, 26], and some showing the connection between mortality and high viral load [27]. Prolonged viral shedding and unsuccessful viral clearance also seems to be linked with disease severity in patients with comorbidities [26]. Interestingly, detection of SARS-CoV-2 in serum was also associated with mortality [28], similarly to what we have shown in the described case. Studies exploring viral dynamics in patients with and without cancer demonstrated that patients with hematological malignancies had the highest viral loads. Moreover, the patients who had received treatment for these malignancies had even higher viral loads [29], coinciding with our investigations of this case.

## Conclusions

This case is an excellent illustration of how the presence of an oncological disease and the treatment for it combined with immunosenescence and inflamm-aging lowers the capabilities of the immune system by impairing humoral immunity and viral clearance and therefore leads to the wide tissue distribution of SARS-CoV-2, harboring detectable SARS-CoV-2 outside the respiratory tract—in blood plasma, PBMCs, urine and feces, which in turn could further exacerbate the disease and lead to a greater fatality risk. The obtained results and the course of the disease once again highlights the need to monitor COVID-19 patients of advanced age and immunocompromised state closely.

## Data Availability

The datasets used and/or analyzed during the current study are available from the corresponding author upon reasonable request.

## Abbreviations

ALAT: Alanine aminotransferase
ASAT: Aspartate aminotransferase
COVID-19: Coronavirus disease 2019
CRP: C-reactive protein
GFR: Glomerular filtration rate
GGT: Gamma-glutamyl transferase
GM-CSF: Granulocyte-macrophage colony-stimulating factor
HIV: Human immunodeficiency virus
IFN-γ: Interferon gamma
IL-6: Interleukin 6
IL-17A: Interleukin 17A
IL-18: Interleukin 18
IL-1β: Interleukin 1β
IL-6: Interleukin 6
IL-8: Interleukin 8
IP-10: Interferon gamma-induced protein 10
LCID: Latvian Centre of Infectious Diseases
MCP-1: Monocyte chemotactic protein 1
MDRD: Modification of diet in renal disease equation
MIP-1α: Macrophage inflammatory protein-1 alpha
NCP: Nucleocapsid protein
PBMC: Peripheral blood mononuclear cells
PCR: Polymerase chain reaction
PCT: Procalcitonin
PDGF-AB/BB: Platelet-derived growth factor
RNA: Ribonucleic acid
S1: Spike protein subunit 1
SARS-COV-2: Severe acute respiratory syndrome coronavirus 2
TNF-α: Tumor necrosis factor-alpha
VEGF-A: Vascular endothelial growth factor A
